# Rare epidermal growth factor receptor (EGFR) mutations in non-small cell lung cancer

**DOI:** 10.1016/j.semcancer.2019.09.015

**Published:** 2020-04

**Authors:** Peter T. Harrison, Simon Vyse, Paul H. Huang

**Affiliations:** Division of Molecular Pathology, The Institute of Cancer Research, London, SW3 6JB, UK

**Keywords:** EGFR, Lung cancer, Kinase inhibitor, Signal transduction, Targeted therapy

## Abstract

Epidermal growth factor receptor (*EGFR*) mutations are the second most common oncogenic driver event in non-small cell lung cancer (NSCLC). Classical activating mutations (exon 19 deletions and the L858R point mutation) comprise the vast majority of *EGFR* mutations and are well defined as strong predictors for good clinical response to EGFR tyrosine kinase inhibitors (EGFRi). However, low frequency mutations including point mutations, deletions, insertions and duplications occur within exons 18–25 of the *EGFR* gene in NSCLC and are associated with poorer responses to EGFRi. Despite an increased uptake of more sensitive detection methods to identify rare *EGFR* mutations in patients, our understanding of the biology of these rare *EGFR* mutations is poor compared to classical mutations. In particular, clinical data focused on these mutations is lacking due to their rarity and challenges in trial recruitment, resulting in an absence of effective treatment strategies for many low frequency *EGFR* mutations. In this review, we describe the structural and mechanistic features of rare *EGFR* mutations in NSCLC and discuss the preclinical and clinical evidence for EGFRi response for individual rare *EGFR* mutations. We also discuss EGFRi sensitivity for complex *EGFR* mutations, and conclude by offering a perspective on the outstanding questions and future steps required to make advances in the treatment of NSCLC patients that harbour rare *EGFR* mutations.

## 1 Introduction

**Fig. 1:**
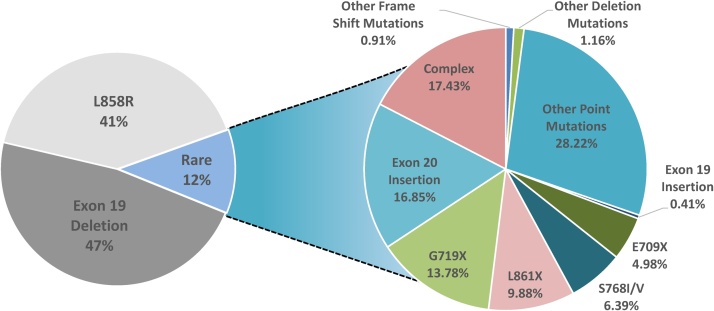
**Pie chart showing the frequencies of EGFR mutations in NSCLC.** Data was acquired from COSMIC databases. Data was filtered to contain only mutations from adenocarcinoma. The common resistance mutations T790M and C797S were filtered out.

**Fig. 2:**
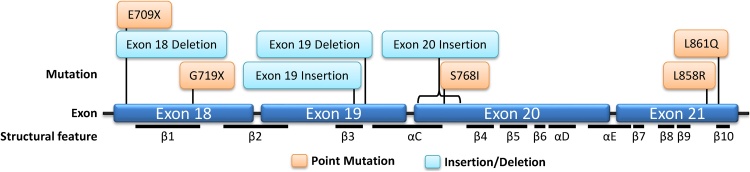
**Lollipop plot showing the position of EGFR mutations and structural features of EGFR.** Orange boxes indicate point mutations, blue boxes indicate insertion/deletion mutations.

Activating mutations in the epidermal growth factor receptor (*EGFR*) gene occur in 10–20% of Caucasian and at least 50% of Asian non-small cell lung cancer (NSCLC) patients [[1], [2], [3], [4]]. Two mutations, deletions in exon 19 and the single amino acid substitution L858R in exon 21, often referred to as “classical” *EGFR* mutations, together account for ˜85% of observed *EGFR* mutations in NSCLC (Fig. 1) and confer sensitivity to EGFR tyrosine kinase inhibitors (EGFRi) [[5], [6], [7]]. Rare mutations account for the remaining ˜15% of *EGFR* mutations in NSCLC and include point mutations, deletions and insertions within exons 18–25 of the *EGFR* gene (Fig. 1, Fig. 2) [8]. Despite being low frequency mutations, given the high prevalence of lung cancer overall, it is estimated that over 30,000 NSCLC patient new diagnoses per year will harbour rare *EGFR* mutations. Therefore, it is crucial to understand the biology of rare *EGFR* mutations and to assess the effectiveness of current treatment options. One obstacle to understanding the differences in biology of rare *EGFR* mutations compared to classical *EGFR* mutations is the lack of patient-derived NSCLC cell line models that harbour endogenous rare *EGFR* mutations. Many preclinical studies thus rely on exogenous expression of rare *EGFR* mutants in model cell lines such as the mouse pro-B cell line Ba/F3 and mouse fibroblast cell line NIH-3T3. Despite these limitations, structural and preclinical data have been used to predict the efficacy of different EGFRi for specific rare *EGFR* mutations. However, there are very few clinical trials that systematically and robustly evaluate the efficacy of EGFRi in NSCLC patients that harbour rare *EGFR* mutations. Due to the paucity of clinical data, the field is largely reliant on pooled post-hoc analyses of clinical trials and case series to evaluate EGFRi response in this heterogeneous group of patients. For example, in 2018 the FDA approved the second-generation EGFRi afatinib for treatment of S768I, L861Q and G719X rare *EGFR* point mutations based on evidence from pooled analysis of three clinical trials, LUX-Lung 2, LUX-Lung 3 and LUX-Lung 6 [9,10]. In the following sections, we describe the structural features of rare *EGFR* mutations and elaborate on the preclinical and clinical evidence for EGFRi response for patients that harbour such rare *EGFR* mutations.

## 2 Classical *EGFR* mutations

### 2.1 Structural and mechanistic features

**Fig. 3:**
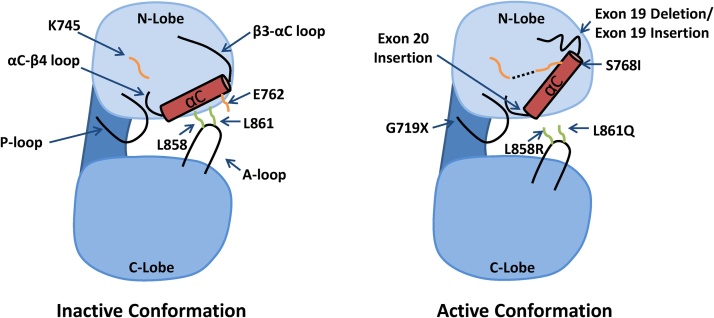
**Cartoon showing the structure of the EGFR kinase domain in the active and inactive conformation.** In the left panel the inactive conformation is shown, and important residues and structural features are labelled. In the right panel the active conformation is shown, and the approximate location of mutations reported in NSCLC are labelled. Dashed lines indicate salt-bridge interactions.

The kinase domain of EGFR has 2 lobes, the N-lobe and the C-lobe, that are separated by the ATP binding cleft. The N-lobe of the EGFR kinase domain is mainly formed of β-strands and the regulatory αC-helix, whereas the larger C-lobe is mainly α-helical and contains the activation loop (A-loop). The inactive state of wild-type (WT) EGFR is characterized by an outward rotation of the αC-helix that is stabilized by a helical turn within the N-terminal portion of the A-loop (Fig. 3) [11]. This conformation prevents the catalytically important salt-bridge interaction between K745 and E762 and is often referred to as the “Src/CDK2-like inactive” conformation due to its similarity to the inactive conformations of Src and CDK2 [12]. K745 and E762 are located in the N-lobe, with E762 positioned on the αC-helix. Together they bind and orientate ATP by forming interactions with the α and β phosphate of ATP respectively. The N-lobe also contains the glycine-rich phosphate binding loop (P-loop) while the C-lobe comprises the DFG motif, the catalytic loop, and the catalytic base.

Structural studies have shown that L858R and exon 19 deletions (Ex19Del) destabilize the inactive conformation of the receptor, leading increased receptor dimerization and activity compared to WT [11,13,14]. L858 lies within the helical turn of the activation loop and forms crucial hydrophobic interactions with residues in the N-lobe when the receptor is in the inactive conformation (Fig. 3). Owing to arginine’s much larger side chain compared to leucine, the L858R substitution is incompatible with the inactive conformation and therefore “locks” the kinase domain in a constitutively active conformation. When in the active conformation, the positively charged L858R is surrounded by a cluster of negatively charged residues (E758, D855, and D837) that further stabilize this configuration [12]. Ex19Del is also thought to destabilize the inactive conformation through shortening the β3-αC loop that prevents the outward rotation of the αC-helix (Fig. 3) [12,14].

### 2.2 Clinical response to EGFRi

First-generation EGFRi are ATP-competitive, reversible inhibitors that preferentially bind to classical EGFR mutant receptors over WT EGFR. L858R and Ex19Del mutant EGFR have a lower affinity for ATP compared to WT EGFR, enabling first-generation EGFRi to outcompete ATP and thus prevent receptor activation [11,15]. This differential affinity gives the inhibitors a large therapeutic window, with gefitinib showing a ˜100 fold increase in potency against L858R compared to WT. This increased potency translates into a 72% response rate (RR) and nearly 10 months median progression-free survival (PFS) in patients with these mutations when treated with first-generation EGFRi [11,[16], [17], [18]]. The most common resistance mechanism to first-generation EGFRi is the gatekeeper mutation T790M, occurring in ˜60% of patients who acquired resistance to first-generation EGFRi [19]. This mutation increases the ATP affinity of the receptor and sterically hinders drug binding, preventing first-generation EGFRi from outcompeting ATP and resulting in activation of the receptor despite EGFRi treatment [20]. Second-generation EGFRi were developed to address the issue of acquired resistance to first-generation EGFRi. This class of compounds binds irreversibly to EGFR at the C797 residue by covalent adduct formation. After forming a covalent, irreversible complex the competitive equilibrium with ATP is lost, overcoming drug resistance caused by the T790M mutation which possesses similar ATP affinity to WT EGFR. Despite promising preclinical results, second-generation EGFRi largely failed in clinical trials evaluating patients with T790M-mediated first-generation EGFRi resistance [[21], [22], [23], [24]]. This failure is due to poor selectivity of the drug for L858R/T790M or Ex19Del/T790M EGFR mutants over WT EGFR, leading to dose-limiting toxicities which limits the clinical efficacy of these drugs in this setting [[25], [26], [27]]. Third-generation EGFRi also bind irreversibly to EGFR by covalent adduct formation. However, in contrast to second-generation EGFRi, third-generation EGFRi show greater selectivity for EGFR T790M mutations over WT EGFR compared to second-generation EGFRi [28]. This greater selectivity has provided a therapeutic window that resulted in rapid clinical success for osimertinib, with pooled analyses of the AURA extension and AURA2 clinical trials identifying a response rate (RR) of 66% and median PFS of 9.9 months in T790M-positive NSCLC patients [29]. This data is comparable to the effect of first-generation EGFRi in patients with classical *EGFR* mutations. The FDA granted accelerated approval for osimertinib as a second-line treatment for EGFR T790M mutant-positive patients following progression on erlotinib or gefitinib in 2015 and regular approval for this indication in 2017 [30]. Interestingly, recent clinical data from the FLAURA trial has also shown that that osimertinib may be superior to first-generation EGFRi in the first-line setting. Osimertinib provided superior median PFS of 17.2 months compared to 8.5 months for gefitinib or erlotinib in treatment-naïve NSCLC patients, resulting in FDA approval to treat patients with classical *EGFR* mutations with osimertinib as a first-line therapy in 2018 [31].

For further literature about EGFRi in NSCLC with classical *EGFR* mutations, the reader is directed to several excellent reviews on the subject [7,14,32,33]. The remainder of this review will discuss the preclinical and clinical studies that have focused on NSCLC with rare *EGFR* mutations.

## 3 Rare *EGFR* exon 18 mutations

### 3.1 E709X and exon 18 deletions

E709 is located in the N-lobe, on the N-terminal side of the β1 strand that immediately precedes the phosphate binding loop (Fig. 2). Mutations at E709 are comprised of the deletion delE709-T710insD (also referred to as exon 18 deletion) and substitutions of E709 for A, G, K, or V, of which E709K is the most common [34,35]. Together, these mutations are reported to account for ˜1.5% of rare *EGFR* mutations in NSCLC, although the actual frequency of E709X and exon 18 deletions may be underreported as they are not detectable by many common commercially available diagnostic kits [35,36]. While delE709-T710insD has been reported as a sole *EGFR* mutation, over 75% of substitutions at E709 are reported as “complex mutations” with the presence of additional *EGFR* mutations such as L858R, Ex19Del, or G719X detected within the same tumour [35,37].

#### 3.1.1 Preclinical data

To investigate E709X mutations, Kobayashi et al. expressed E709K and delE709-T710insD in Ba/F3 and NIH-3T3 cells [34]. Ba/F3 cells expressing E709X mutations were shown to grow in the absence of IL-3 while NIH-3T3 cells expressing the mutant constructs formed foci in a focus formation assay, suggesting that these mutations are oncogenic drivers. To examine the EGFRi sensitivity of these mutations, the authors performed dose-response experiments on the Ba/F3 cells using 7 different EGFRi; first-generation inhibitors gefitinib and erlotinib, second-generation inhibitors afatinib, dacomitinib and neratinib, and third-generation inhibitors osimertinib and rociletinib. They found that both E709K and delE709-T710insD were significantly less sensitive to gefitinib, erlotinib, and osimertinib compared to Ba/F3 cells expressing Ex19Del, with delE709-T710insD showing the greatest resistance to these inhibitors. However, E709K and delE709-T710insD showed sensitivity to the second-generation inhibitors afatinib and neratinib comparable to that of Ex19Del. In parallel, western blot analysis of HEK293 cells expressing E709K, delE709-T710insD, and Ex19Del revealed that the tyrosine phosphorylation of EGFR was inhibited in each cell line following treatment with afatinib or neratinib. In contrast, only Ex19Del showed EGFR inhibition following treatment with the same dose of gefitinib, erlotinib, or osimertinib, indicating that second-generation EGFRi have the greatest affinity for rare E709X and exon 18 deletion mutant EGFR compared to first- or third-generation inhibitors

#### 3.1.2 Clinical data

The results of trials which have evaluated outcomes of NSCLC patients with rare *EGFR* mutations in exon 18 are summarised in Table 1. As a result of the low incidence of E709X substitution and exon 18 deletion mutations, very little is known about the clinical response of these mutations to EGFRi. Kobayashi et al. reported 53% RR across 15 patients with E709X complex mutations and 25% RR in 4 delE709_T710insD patients treated with first-generation EGFRi [8]. This data supports the preclinical evidence that identified substitution mutations at E709 as more sensitive to first-generation EGFRi overall compared to exon 18 deletions [34]. A report assessing the use of afatinib identified drug activity with time to treatment failure (TTF) > 12 months in 4 patients with E709X mutations [38], however all patients had complex mutations with either additional L858R or G719X *EGFR* mutations. Further clinical studies will be required to validate preclinical data that second-generation EGFRi are more effective for E709X mutations, which in turn may depend on uptake of diagnostic tests that can detect these rare *EGFR* variants.

**Table 1:** Clinical outcomes of NSCLC patients with rare *EGFR* mutations in exon 18 after EGFRi treatment.

Mutation	Study (Reference)	*EGFR* mutation(s), *n* treated with EGFRi	EGFRi used	ORR (%)	DCR (%)	Median PFS,	Median OS,
				(CR + PR)	(CR + PR + SD)	months (95% CI)	months (95% CI)
E709X	Wu & Shih [35]	DelE709-T710insD, *n = 5*	Gefitinib / Erlotinib	50.0%	72.2%	6.2	29.3
		E709X complex mutations, *n = 13*					
	Kobayashi & Mitsudomi [8]	DelE709_T710insD, *n = 4*	Gefitinib / Erlotinib	25.0%	50.0%	NR	NR
		E709X complex mutations, *n = 15*		53.0%	86.7%	NR	NR
G719X	Chiu et al. [39]	G719X, *n = 78*	Gefitinib / Erlotinib	36.8%	72.4%	6.3	NR
		G719X + L861Q, *n = 9*		88.9%	100.0%	11.9	NR
		G719X + S768I, *n = 10*		50.0%	100.0%		
	Kobayashi & Mitsudomi [8]	G719X, *n = 148*	Gefitinib / Erlotinib	65.5%	32.0%	NR	NR
		G719X complex, *n = 58*		59.0%	89.7%	NR	NR
	Kate et al. [108]	G719X, *n = 5*	Gefitinib / Erlotinib	50.0%	NR	9.0 (NE)	NR
	Xu et al. [40]	G719X, *n = 14*	Gefitinib / Erlotinib / Icotinib	42.9%	78.6%	5.98 (1.53 - 10.42)	19.81 (16.81 - 22.81)
	Sequist et al. [21]	G719X, *n = 4*	Neratinib	75.0%	100.0%	12.1	NR
	Yang et al. [9]	G719X single, *n = 8*, + G719X complex, *n = *6	Afatinib	77.8%	NR	13.8 (6.8 - NE)	26.9 (16.4 - NE)
	Ahn et al. [112]	G719X *n = 19*	Osimertinib	52.6%	NR	NR	NR
Pooled	Beau-Faller et al. [41]	**Total n = 18:** G719X, *n = 14,* E709X, *n = 2,*	Gefitinib / Erlotinib	7.0%	33.3%	3 (1 - NE)	22 (1 - 44)
		rare exon 18 substitutions, *n = 2*					
	Passaro et al. [113]	**Total n = 42:** G719X, *n = 35,* E709X, *n = 3,*	Gefitinib / Erlotinib / Afatinib	31.0%	69.0%	8.3 (4.8 - 11.7)	17 (8.2 - 25.7)
		not specified*, n = 4*					

Legend: EGFRi, EGFR inhibitor; ORR, objective response rate; CR, complete response; PR, partial response; DCR, disease control rate; SD, stable disease; PFS, progression-free survival; CI, confidence interval; OS, overall survival; NR, not reported; NE, not estimable.

### 3.2 G719X

Among rare *EGFR* mutations in NSCLC, G719X substitutions (which include G719S, G719A, G719C and G719D substitutions) are one of the more commonly observed mutations second only to exon 20 insertions and represent approximately 1.5–3% of all *EGFR* mutations in NSCLC [8]. G719X mutations can occur as independent *EGFR* mutations or may be present in combination with additional point mutations such as S768I or L819Q as complex *EGFR* mutations [41].

#### 3.2.1 Preclinical data

G719 is located in the phosphate binding “P-loop” within the N-lobe (Fig. 3), which participates in the coordination of ATP by arching over the triphosphate moiety. The P-loop also contributes to a set of hydrophobic interactions that hold the αC-helix in the inactive conformation. P723, which lies at the C-terminal end of the P-loop, packs together with L747 and L862 to form a hydrophobic cluster surrounding L858 [11]. In this conformation, a glycine at position 719 is favoured. Substitution of glycine for serine at 719 reduces the flexibility of the P-loop and weakens the hydrophobic interactions that hold the αC-helix in the inactive conformation, leading to a 10-fold increase in kinase activity compared to WT EGFR [11,12]. Shan et al. predict that the structural changes induced by G719S mutation also increase propensity for dimerization and subsequent activation of EGFR in a similar fashion to the L858R classical mutation. Substitution of G719 for A, C, and D has also been reported in NSCLC [39]. Yun et al. suggest that substitution for any non-glycine residue at position 719 would weaken the hydrophobic interactions that hold the αC-helix in the inactive conformation [11].

Kobayashi et al. showed that Ba/F3 cells expressing G719A mutations were more sensitive to second-generation EGFRi than first-generation EGFRi *in vitro* [34]. They found that G719A showed poor response to gefitinib, erlotinib, and osimertinib when compared with cells expressing Ex19Del. However, they showed that cells expressing G719A were sensitive to the second-generation EGFRi afatinib and neratinib, with IC90 s of 0.9 nM and 1.1 nM respectively. Western blot analysis showed that this difference in sensitivity to EGFRi correlated to a decrease in EGFR phosphorylation following drug treatment. When treated with up to 1 μM of first-generation or third-generation EGFRi G719A cells retained EGFR phosphorylation. However, when treated with just 10 nM of afatinib, the G719A mutant cells almost entirely lost EGFR phosphorylation.

#### 3.2.2 Clinical data

In one of the largest clinical studies focused on uncommon *EGFR* mutations, Chiu et al. assessed the effectiveness of gefitinib and erlotinib in 78 patients with single G719X mutations compared to common Ex19Del and L858R mutations [39]. Although patients with single G719X mutations were sensitive to EGFRi treatment (overall response rate (ORR) = 36.8%, disease control rate (DCR) = 72.4%, n = 78), they were markedly less sensitive when compared to L858R mutations (ORR = 67.5%, DCR = 95.6%, n = 256) or Ex19Del (ORR = 65.3%, DCR = 94.5%, n = 222). These findings were coupled with poorer patient outcomes, with a median PFS of 6.3 months for G719X patients compared to a median PFS of between 10–13 months for L858R and Ex19Del patients. In line with preclinical studies, second-generation EGFRi have been shown to be more effective than first-generation inhibitors in G719X patients [34]. In an early phase II clinical trial for neratinib (NCT00266877), despite disappointing results for *EGFR* mutant lung cancer overall, three out of four patients with the rare *EGFR* mutation G719X showed a partial response to neratinib, whilst the fourth exhibited stable disease for 40 weeks [21]. However, severe dose-limiting toxicities for neratinib were observed during this trial which halted further investigation of neratinib for *EGFR-*mutant NSCLC. G719X mutations have also been shown to be responsive to the second-generation EGFRi afatinib. A post-hoc analysis of data from 32 patients pooled from LUX-Lung 2, LUX-Lung 3 and LUX-Lung 6 trials revealed clinical activity of afatinib against rare EGFR mutant (G719X, S786I, L861Q) lung cancers [9]. Across 8 patients harbouring single G719X mutations and 6 with complex G719X mutations, afatinib treatment resulted in 77.8% RR and 13.8 months PFS (Table 1), which in January 2018 led the FDA to broaden the indication for afatinib to include NSCLC patients that harbour G719X mutations [10]. A recent phase II trial has suggested that the third-generation inhibitor osimertinib may also have clinical activity in this patient population, with 52.6% RR reported in 19 patients harbouring G179X mutations [112]. However, further trials will be required to determine whether osimertinib can provide significant survival benefits compared to afatinib treatment.

## 4 Rare *EGFR* exon 19 mutations

### 4.1 Exon 19 insertion

Low prevalence exon 19 insertions have been reported that account for approximately 2% of exon 19 aberrations and 1% of all *EGFR* mutations in NSCLC [42]. Exon 19 insertions are characterised by 18 base pair insertions that result in the addition of a 6 amino acid sequence that, in the majority of cases, begins at codon 744 or 745 of the *EGFR* gene. Although the exact amino acid sequence inserted is heterogeneous, the 4 amino acid sequence PVAI is shared and common to all reported exon 19 insertions.

#### 4.1.1 Preclinical data

Despite nuanced differences in the exact sequence of exon 19 insertions mutations, there are 2 conserved effects on the 3D structure; the addition of the 6-residue sequence to the loop that connects the β3-strand to the αC-helix (Fig. 3), and the alteration of E746 and L747, the last 2 residues of the β3-strand in WT EGFR. Insertion of the 6-residue sequence is likely to be well tolerated as this loop is flexible in the structure of WT EGFR. The shift in amino acids that alters the identity of the E746 residue following insertion of a 6-residue sequence is also likely to have little structural effect as it is exposed on the surface of the kinase. By contrast, the L747 residue participates in a crucial hydrophobic core that stabilizes the inactive form of the kinase domain. He et al. report 11 patients harbouring exon 19 insertions and in each patient a common effect of the insertion is that the identity of the leucine residue at position 747 in WT EGFR is changed to a proline residue at that position (L747P). L747P is predicted to disfavour the formation of this hydrophobic core and thereby lead to constitutive activation of EGFR; a mechanism analogous to that of the L858R substitution, which lies immediately adjacent to L747 in the hydrophobic core.

To assess the sensitivity of exon 19 insertions mutations to EGFRi, He et al. expressed 2 different exon 19 insertions (I744_K745insKIPVAI and K745_E746insTPVAIK) constructs in Ba/F3 cells [42]. Dose-response experiments revealed that these exon 19 insertions showed a similar sensitivity to gefitinib and afatinib compared to Ba/F3s expressing Ex19Del. Western blot analysis of NIH-3T3 cells expressing exon 19 insertions revealed a loss of EGFR phosphorylation following gefitinib or afatinib treatment comparable to that observed in Ex19Del expressing cells. This data led the authors to conclude that exon 19 insertions mutations are EGFRi sensitive and recommends that NSCLC patients harbouring these mutations should be treated with EGFRi.

#### 4.1.2 Clinical data

Similar to E709 deletions and point mutations, exon 19 insertions are not commonly screened as part of routine diagnosis and therefore clinical data regarding EGFRi sensitivity is extremely limited. In a small case report, He et al. reported that 3 out of 4 patients with metastatic NSCLC harbouring exon 19 insertions showed a partial response to EGFRi, providing evidence to suggest exon 19 insertions may confer EGFRi sensitivity similar to classical *EGFR* mutations [42]. Several other case studies have reported partial responses or stable disease for small numbers of exon 19 insertion patients treated with first-generation EGFRi, however the durability of response has been extremely variable, ranging from a relatively short PFS of 5.9 months to a much longer 24 months [8,[43], [44], [45]] (Table 2). This preliminary data suggests that exon 19 insertions could be considered EGFRi sensitive in the clinical setting, however larger patient cohorts will be required to refine which specific EGFRi provides effective and durable responses.

**Table 2:** Clinical outcomes of NSCLC patients with rare *EGFR* mutations in exon 19 after EGFRi treatment.

Mutation	Study (Reference)	*EGFR* mutation(s), *n* treated with EGFRi	EGFRi used	ORR (%)	DCR (%)	Median PFS,	Median OS,
				(CR + PR)	(CR + PR + SD)	months (95% CI)	months (95% CI)
Ex 19 insertion	Kobayashi & Mitsudomi [8]	Exon 19 insertion, *n = 10*	Gefitinib / Erlotinib	40%	100%	NR	NR
	He et al. [42]	p.K745_E746insIPVAIK, *n = 1*	Afatinib	PR		14	NR
		p.K745_E746insTPVAIK, *n = 1*	Erlotinib	PR		19	NR
		p.I744_K745insKIPVAI, *n = 1*	Erlotinib	PR		50	NR
		Unspecified exon 19 insertion, *n = 1*	XL674	NR		4	NR
	Park et al. [114]	p.I744_K745insKIPVAI, *n = 1*	Gefitinib	PR		4.2	NR
		p.I744_K745insKIPVAI, *n = 1*		SD		4.9	NR
		p.I744_K745insKIPVAI, *n = 1*		PR		8.8	NR
	Iyevleva et al. [115]	p.I744_K745insKIPVAI, *n = 1*	Gefitinib	PR		5	NR
		p.I744_K745insKIPVAI, *n = 1*		SD		11+	NR
		p.I744_K745insKIPVAI, *n = 1*		SD		9+	NR

Legend: EGFRi, EGFR inhibitor; ORR, objective response rate; CR, complete response; PR, partial response; DCR, disease control rate; SD, stable disease; PFS, progression-free survival; CI, confidence interval; OS, overall survival; NR, not reported; +, continued SD at date of last follow-up.

## 5 Rare *EGFR* exon 20 mutations

### 5.1 Exon 20 insertions

*EGFR* exon 20 insertion mutations encompass a heterogeneous range of insertions of between 1–7 amino acids that occur towards the C-terminal end of the αC-helix (Fig. 2, Fig. 3) [46]. After classical mutations, *EGFR* exon 20 insertions are the next most common *EGFR* mutation in NSCLC, with frequencies reported at between 4–10% of all observed *EGFR* mutations [[46], [47], [48]].

#### 5.1.1 Preclinical data

Crystal structures of EGFR exon 20 insertion mutants have shown that the insertion forms a wedge that “pushes” the αC-helix and prevents its outward rotation into the inactive conformation, leading to constitutive activation of the kinase domain [14,46]. Different EGFR exon 20 insertion mutations display different sensitivity to EGFRi. Yasuda et al. found that A763_764insFQEA has a high affinity for first-generation EGFRi gefitinib, whereas D770_N771insNPG did not. *In vitro* kinetic studies revealed that D770_N771insNPG was able to activate EGFR whilst retaining its ATP affinity, preventing competitive binding of gefitinib [46]. Additionally, computational modelling has shown that exon 20 insertions cause significant structural alterations in the P-loop and αC-helix that result in a relatively small drug binding pocket that sterically hinders first-generation EGFRi binding [49]. Conversely, computational modelling of A763_764insFQEA suggests that insertions that occur within the αC-helix, as opposed to within the loop following the αC-helix, may have an activation mechanism more similar to that of Ex19Del and L858R, and therefore would be predicted to be sensitive to first-generation EGFRi [46].

A preclinical study utilized the Ba/F3 and NIH-3T3 model systems to assess the sensitivity of 5 exon 20 insertion mutations to second-generation EGFRi and noted that dacomitinib was particularly effective for insertions that included a glycine residue at position 770 [50]. Dose-response experiments showed that only cells expressing the D770delinsGY mutation were sensitive to a low enough *in vitro* dose of dacomitinib (17.5  nM) that would predict drug sensitivity in patients, based on the plasma concentrations of dacomitinib achieved after a standard dosing regimen of 45 mg once per day in a phase II trial [51].

The preclinical evidence to support the use of third-generation EGFRi for exon 20 insertions remains unclear. Third-generation EGFRi osimertinib and rociletinib have shown poor responses in a study of two lung cancer patient-derived xenograft models harbouring P772_H773insDNP and H773_V774insNPH exon 20 insertion mutations [52]. However, another study has shown significant anti-tumour activity of osimertinib in xenograft models of the WT EGFR lung cancer cell line H2073 cells engineered to express either D770_N771insSVD or V769_D770InsASV insertions using CRISPR [53]. While osimertinib may not be effective for all *EGFR* exon 20 insertions, it is possible that the sensitivity to third-generation EGFRi may be variable due to the heterogeneity of the exact location and size of specific mutations.

Recently, EGFRi with the capacity to target exon 20 insertion mutations have been evaluated, of which poziotinib is the most clinically advanced. Poziotinib differs structurally from other EGFRi; it has a flexible quinazoline core and small linking groups that, based on 3D modelling, has been predicted to bind tightly to the restricted drug-binding pocket of exon 20 insertion EGFR kinases [49]. Robichaux et al. expressed 7 different *EGFR* exon 20 insertion mutations in Ba/F3 cells. Dose-response experiments showed that cells expressing *EGFR* exon 20 insertion mutations were sensitive to low doses of poziotinib and concomitantly that mutant EGFR phosphorylation was potently reduced following poziotinib treatment [49].

In addition to poziotinib, several other compounds that inhibit exon 20 insertion mutations are being investigated in preclinical studies. TAK-788 is an irreversible inhibitor that selectively targets exon 20 insertion mutations. In preclinical studies utilizing the Ba/F3 model system, Gonzalvez et al. showed TAK-788 to be effective against 14 different exon 20 insertion mutations [54]. The authors also showed tumour regression in a patient-derived xenograft NSCLC model harbouring an exon 20 insertion mutation following treatment with TAK-788. TAS6417 is a covalent inhibitor that binds irreversibly to C797 in the ATP binding site of the exon 20 insertion mutant kinase [55]. Cell free *in vitro* kinase assays showed that TAS6417 has selectivity for D770_N771insNPG over WT EGFR. Western blot analysis found that TAS6417 was able to inhibit EGFR phosphorylation in NIH-3T3 cell lines expressing 6 different exon 20 insertion mutations. In the absence of a cell line model harbouring an endogenous EGFR exon 20 insertion, Hasako et al. employed transcription activator-like effector nuclease (TALEN) mutagenesis to engineer a model based on the H1975 *EGFR* mutant L858R/T790M cell line. In a two-step process, TALEN was first used to introduce D770_N771insSVD into H1975 cells and subsequently, the endogenous L858R/T790M EGFR mutation was deleted. TAS6417 was shown to inhibit cell growth of the H1975 *EGFR* D770_N771insSVD mutant cell line *in vitro* and *in vivo*. Western blot analysis of tissue collected from mice bearing H1975 *EGFR* D770_N771insSVD tumours revealed that TAS6417 is able to inhibit EGFR phosphorylation within the tumour, whilst having minimal effect on WT EGFR phosphorylation within the skin tissue. Another study has identified Compound 1A, a covalent inhibitor that also binds irreversibly to C797 and may be effective against EGFR exon 20 insertions [56]. The structure of compound 1A is based on the original pyrimidine core of osimertinib, but unlike the third-generation EGFRi, this novel drug possesses additional groups that form more extensive interactions with a deep hydrophobic pocket of the EGFR kinase. Compound 1A has been shown to potently inhibit phosphorylation of EGFR in Ba/F3 cells expressing exon 20 insertion mutations and has shown anti-proliferative effects against a patient-derived NSCLC cell line harbouring an *EGFR* P772_H773insPNP mutation. Currently, the use of compound 1A *in vivo* is likely to be limited by poor oral bioavailability (11%) and short half-life (0.5 h), however, further development may yield a compound with improved pharmacokinetic properties that utilises this mechanism to target EGFR exon 20 insertion kinases.

#### 5.1.2 Clinical data

Clinical trials which have evaluated EGFRi in NSCLC patients with rare *EGFR* mutations in exon 20 are summarised in Table 3. Clinical studies have revealed notable differences in EGFRi sensitivity for distinct types of exon 20 insertions within this heterogeneous group of mutations that supports preclinical observations. The majority of NSCLC patients harbouring *EGFR* exon 20 insertions are resistant to first- and second-generation EGFRi, with low response rates of between 0–27% reported [9,21,41,57]. However, as predicted by structural data and kinetic analysis of drug binding *in vitro*, insertions that occur in the portion of the *EGFR* gene which encodes the αC-helix, as opposed to within the loop that immediately follows, are the exception to the rule [46]. Multiple reports have demonstrated partial responses to erlotinib for patients harbouring the A763_Y764insFQEA insertion, which displays similar sensitivity to first-generation EGFRi as classical *EGFR* mutations [47,57,58]. Although currently licensed EGFRi have not shown any significant benefit for patients with other *EGFR* exon 20 insertions, emerging EGFRi including poziotinib, TAK-788 and tarloxotinib are in clinical development with promising activity for this subgroup of NSCLC patients. Further details of these drugs have been recently reviewed in depth by our laboratory elsewhere [59].

**Table 3:** Clinical outcomes of NSCLC patients with rare *EGFR* mutations in exon 20 after EGFRi treatment.

Mutation	Study (Reference)	*EGFR* mutation(s), *n* treated with EGFRi	EGFRi used	ORR (%)	DCR (%)	Median PFS,	Median OS,
				(CR + PR)	(CR + PR + SD)	months (95% CI)	months (95% CI)
Ex 20 insertion	Beau-Faller et al. [41]	Multiple exon 20 insertions, *n = 19*	Gefitinib / Erlotinib	5.0%	36.0%	2 (1 - NE)	9.5 (4 - 15)
	Naidoo et al. [57]	Multiple exon 20 insertions, *n = 11*	Erlotinib	27.0%	45.0%	2.5	26
	Xu et al. [40]	Multiple exon 20 insertions, *n = 12*	Gefitinib / Erlotinib / Icotinib	8.3%	58.3%	2.00 (0.00 - 5.41)	16.69 (13.93 - 19.45)
	Kate et al. [108]	Multiple exon 20 insertions, *n = 7*	Gefitinib / Erlotinib / Afatinib / Osimertinib	0.0%	0.0%	1.9 (0.3-3.5)	NR
	Jänne et al. [109]	Multiple exon 20 insertions, *n = 5*	Dacomitinib	20.0%; 1 PR in D770delinsGY patient	40.0%	12.4 months PFS in D770delinsGY patient	NR
	Yang et al. [9]	Multiple exon 20 insertions, *n = 23*	Afatinib	8.7%	65.0%	2.7 (1.8 - 4.2)	9.2 (4.1 - 14.2)
	Sequist et al. [21]	Multiple exon 20 insertions, *n = 3*	Neratinib	0.0%	NR	NR	NR
	Robichaux et al. [49]	Multiple exon 20 insertions, *n = 44*	Poziotinib	43.0% confirmed	NR	5.6 (5.06 - NE)	NR
	Jänne et al. [110]	Multiple exon 20 insertions, *n = 26*	TAK-788	54.0%	89.0%	NR	NR
S768I	Leventakos et al. [60]	S768I, *n = 1*	Erlotinib	PD		3	5
		S768I complex, *n = 3*		33.3%	100.0%	6 - 30 months	23 - 51 months
	Kobayashi & Mitsudomi [8]	S768I, *n = 12*	Gefitinib / Erlotinib	42.0%	58.0%	NR	NR
		S768I complex, *n = 18*		53.0%	94.0%	NR	NR
	Chiu et al. [39]	S768I, *n = 6*	Gefitinib / Erlotinib	33.3%	66.7%	NR	NR
		S768I + G719X, *n = 10*		50.0%	100.0%	NR	NR
	Kate et al. [108]	S768I, *n = 2*	Erlotinib	0.0%	0.0%	1.0 (NE)	NR
	Chen et al. [111]	S768I, *n = 3*, S768I + L858R, *n = 4,*	Gefitinib / Erlotinib / Icotinib	20.0%	70.0%	2.7 (NR)	14.5 (NR)
		*S768I + ex19del, n = 3*					
	Yang et al. [9]	S768I single, *n = 1,* + S768I complex, *n = 7*	Afatinib	100.0%	100.0%	14.7 (2.6 - NE)	NE (3.4 - NE)
	Ahn et al. [112]	S768I *n = 8*	Osimertinib	37.5%	NR	NR	NR

Legend: EGFRi, EGFR inhibitor; ORR, objective response rate; CR, complete response; PR, partial response; DCR, disease control rate; SD, stable disease; PD, progressive disease; PFS, progression-free survival; CI, confidence interval; OS, overall survival; NR, not reported; NE, not estimable.

### 5.2 S768I

In addition to exon 20 insertions, the point mutation S768I occurs in the region that encodes the αC-helix in exon 20 of the *EGFR* gene (Fig. 3) and has been reported at a frequency of between 0.6–1% of *EGFR* mutations in NSCLC [8,60]

#### 5.2.1 Preclinical data

Molecular dynamics simulations have shown that S768I stabilizes an active αC-in conformation by improving the hydrophobic packing between the αC helix and the adjacent β9 strand [12]. Banno et al. found that Ba/F3 cells expressing EGFR S768I were less sensitive to first-generation and third-generation EGFRi compared to the L858R mutation [61]. Western blot analysis revealed that S768I expressing Ba/F3s retained EGFR phosphorylation following treatment with 100 nM of erlotinib or osimertinib, whereas L858R expressing cells had reduced EGFR phosphorylation under the same conditions. However, S768I expressing cells did possess sensitivity to afatinib that was comparable with L858R, and treatment with 10 nM afatinib was sufficient to reduce S768I EGFR phosphorylation. Although no NSCLC lines harbouring endogenous S768I are commercially available, Bann et al. studied the oesophageal cancer cell line KYSE540 that expresses harbour endogenous S768I [61]. Dose-response experiments and western blot analysis of this cell line showed comparable results to the Ba/F3 model system, providing further evidence that S768I is resistant to first-generation and third-generation EGFRi compared to L858R, but sensitive to the second-generation EGFRi afatinib.

#### 5.2.2 Clinical data

Whilst the S768I mutation can occur in isolation, it is more common that S768I co-occurs with additional point mutations in *EGFR* to form complex mutations, which can confound interpretation of the clinical data. For example, large variation in responses to first-generation EGFRi have been reported across multiple case studies of S768I mutant patients, ranging from no response and progressive disease [62,63], to good partial responses and intermediate sensitivity [60,[64], [65], [66]]. Highlighting the heterogeneity of durability of response, Leventakos et al. reported a wide range of 3–30 months PFS across 9 patients with either isolated S768I mutation or S768I/G719X or S768I/L858R complex mutations [60]. The low frequency of these mutations and the variations in co-occurring *EGFR* point mutations present a significant challenge in dissecting the reasons behind these inconsistencies in the clinical data. Interestingly, Chiu et al. observed that the complex mutation S768I/G719X may be more sensitive to first-generation EGFRi treatment compared to the single S768I point mutation alone, with 50% RR (n = 10) for complex S768I mutations vs. 33% RR (n = 7) for single S768I mutation [39]. Preclinical data suggested the second-generation inhibitor afatinib may be more effective than first-generation inhibitors for S768I mutant EGFR [61]. In the post-hoc analysis of the LUX-Lung 2, LUX-Lung 3 and LUX-Lung 6 trials, the second-generation EGFRi afatinib achieved 100% ORR and 14.7 months median PFS for 8 patients with S768I mutant NSCLC leading to FDA approval of this drug for NSCLC patients with EGFR S768I mutations [9]. Notably however, within this analysis, 7 out of 8 of the S768I mutant patients harboured complex mutations with either G719X or L858R mutations and therefore it remains unclear whether afatinib will demonstrate consistent responses for patients with single S768I point mutations without studies in larger cohorts.

## 6 Rare *EGFR* exon 21 mutations

### 6.1 L861Q

L861Q is located within the activation loop of EGFR (Fig. 3) and accounts for around 3% of *EGFR* mutations in NSCLC [61,67].

#### 6.1.1 Preclinical data

Molecular dynamics simulations have shown that L861Q stabilizes an active αC-in conformation through the formation of new H-bonds near the C-terminal of the αC-helix [12]. Studies of L861Q in the Ba/F3 model system demonstrated that similar to S768I mutations, L861Q is resistant to first-generation EGFRi compared to L858R, but unlike S768I, L861Q was sensitive to both afatinib and osimertinib treatment [61]. This correlates with western blot analysis showing that EGFR phosphorylation was retained in cells expressing L861Q following treatment with erlotinib, but was lost following treatment with 10 nM afatinib or 100 nM osimertinib. The oesophageal cancer cell line KYSE270 that harbours an endogenous *EGFR* L861Q mutation also showed comparable results to the Ba/F3 model system. These data suggest that both second- and third-generation inhibitors may be effective to target L861Q mutations.

#### 6.1.2 Clinical data

The results of clinical trials which have evaluated EGFRi in L861Q mutant NSCLC are summarised in Table 4. In the largest cohort reported of 57 patients with L861Q mutations, Chiu et al. observed a 40% RR to first-generation EGFRi, suggesting an intermediate sensitivity comparable to S768I and G719X point mutations [39]. This finding supports preclinical observations that L861Q mutations have a reduced sensitivity to first-generation EGFRi compared to L858R, that is similar to the S768I and G719X mutations [8,61]. Post-hoc analysis of the LUX-lung trials revealed afatinib treatment resulted in a 56% RR and 8.2 months median PFS across 12 patients with single L861Q point mutations, 3 patients with L861Q/G719X, and 1 patient with L861Q/Ex19Del complex mutations [9]. These data support preclinical evidence that L861Q mutations are sensitive to afatinib, and led to FDA approval for afatinib for L861Q mutant positive NSCLC [10]. A phase II trial for osimertinib reported partial response in 77.8% of patients with L861Q mutations (*n* = 9) [112]. These data support the preclinical observation that L861Q mutations are sensitive to third-generation EGFRi [61] and suggests that osimertinib may be an effective treatment option for patients harbouring L861Q mutations.

**Table 4:** Clinical outcomes of NSCLC patients with rare *EGFR* mutations in exon 21 after EGFRi treatment.

Mutation	Study (Reference)	*EGFR* mutation(s), *n* treated with EGFRi	EGFRi used	ORR (%)	DCR (%)	Median PFS,	Median OS,
				(CR + PR)	(CR + PR + SD)	months (95% CI)	months (95% CI)
L861Q	Chiu et al. [39]	L861Q, *n = 57*	Gefitinib / Erlotinib	39.6%	75.5%	8.1	NR
		L861Q + G719X, *n = 9*		88.9%	100.0%	NR	NR
	Xu et al. [40]	L861Q, *n = 15*	Gefitinib / Erlotinib / Icotinib	46.7%	80.0%	8.9 (4.47 - 13.34)	21.98 (12.35 - 31.61)
	Kate et al. [108]	L861Q, *n = 2*	Erlotinib	0.0%	0.0%	1.8 (NE)	NR
	Kobayashi & Mitsudomi [8]	L861Q, *n = 64*	Gefitinib / Erlotinib	39.0%	77.0%	NR	NR
		L861Q complex, *n = 12*		92.0%	100.0%	NR	NR
	Yang et al. [9]	L861Q single, *n = 12*, + L861Q complex, *n = 4*	Afatinib	56.3%	NR	8.2 (4.5 - 16.6)	17.1 (15.3 -21.6)
	Ahn et al. [112]	L861Q *n = 9*	Osimertinib	77.8%	NR	NR	NR

Legend: EGFRi, EGFR inhibitor; ORR, objective response rate; CR, complete response; PR, partial response; DCR, disease control rate; SD, stable disease; PFS, progression-free survival; CI, confidence interval; OS, overall survival; NR, not reported; NE, not estimable.

## 7 Other rare *EGFR* mutations

### 7.1 EGFR kinase domain duplication (EGFR-KDD)

EGFR-KDD is most commonly a duplication of exons 18–25 or exons 18–26, which encodes the tyrosine kinase domain, although cases of duplication of exons 14–26 and 17–25 have been reported [68]. EGFR-KDD was first described in NSCLC in 2015 following next generation sequencing of a tumour specimen derived from a patient that displayed durable response to erlotinib despite no detectable common *EGFR* mutations by a polymerase chain reaction-based assay [69]. Although the exact frequency of EGFR-KDDs is uncertain in the absence of routine diagnostic NGS, multicentre studies have reported EGFR-KDDs at 0.2% (n = 1510) [70] and 0.24% (n = 5394) [68] of *EGFR*-mutant NSCLC patients, indicating that duplications may be one of the rarest types of *EGFR* mutation in lung cancer.

#### 7.1.1 Preclinical data

When expressed in NR6, a mouse fibroblast cell line, and Ba/F3 cells EGFR-KDD displayed high levels of constitutive receptor phosphorylation compared to WT EGFR [71]. Gallant et al. also found that A1235 cells, a human glioblastoma cell line which harbours endogenous EGFR-KDD, showed high levels of constitutive EGFR phosphorylation in the presence and absence of serum. Computational modelling revealed that EGFR-KDD are capable of forming intramolecular N-lobe to C-lobe asymmetric dimers, which result in constitutive activation of the receptor [71]. To assess the sensitivity of EGFR-KDD to currently available EGFRi, Gallant et al. treated Ba/F3 cells expressing EGFR-KDD with erlotinib, afatinib, and osimertinib. They found that EGFR-KDD was most sensitive to afatinib. They also showed that phosphorylation of the downstream signalling node ERK decreased following EGFRi treatment, observing similar results in NR6-EGFR-KDD and A1235 cells, indicating that ERK signalling is important for EGFR-KDD-dependent cell survival.

#### 7.1.2 Clinical data

EGFR-KDD has been reported in patients without any additional *EGFR* mutations, supporting the preclinical evidence that duplication of the kinase domain alone can function as an oncogenic driver in NSCLC (Table 5). Although clinical data is limited for this rare mutational event, evidence reported thus far suggests that EGFR-KDD confers sensitivity to targeted EGFRi. In a case report presented by Baik et al., a lung cancer patient harbouring EGFR-KDD demonstrated a durable partial response to second-line treatment with gefitinib for 6 years until disease progression [69]. Following a short regimen of chemotherapy and pemetrexed, which was discontinued due to disease progression, rechallenge with erlotinib in this patient as a fourth-line therapy led to tumour response for a further 3 years. In another case report, Gallant and colleagues identified a NSCLC patient with EGFR-KDD who showed a partial response following 2 cycles of afatinib treatment [71]. Acquired resistance developed after 7 cycles of afatinib, with amplification of EGFR-KDD detected – implicating increase in oncogene dosage by amplification of EGFR-KDD as a mechanism of resistance to afatinib therapy.

**Table 5:** Clinical outcomes of NSCLC patients with EGFR kinase domain duplications after EGFRi treatment.

Mutation	Study (Reference)	EGFR mutation(s), n treated with EGFRi	EGFRi used	Response to EGFRi (PFS)
EGFR-KDD	Baik et al. [69]	EGFR-KDD exons 18-25, n = 1	Gefitinib > Erlotinib	Gefitinib PR (6 years), erlotinib PR (5 years)
	Gallant et al. [71]	EGFR-KDD exons 18-25, n = 1	Afatinib	PR (PFS NR)
	Wang et al. [68]	EGFR-KDD exons 18-25, n = 1	Erlotinib > Osimertinib	Erlotinib PD (2 mo), osimertinib PD (2 mo)
		EGFR-KDD exons 18-25, n = 1	Gefitinib > Afatinib > Osimertinib	Gefitinib PR (5 mo), afatinib PD (2 mo), osimertinib PR (4 mo)
		EGFR-KDD exons 18-25, n = 1	Gefitinib	SD (11 mo)
		EGFR-KDD exons 18-25, n = 1	Icotinib + Apatinib	PR (4+ mo - PFS not reached)
		EGFR-KDD exons 18-25, n = 1	Gefitinib > Erlotinib	Gefitinib PD (3 mo), erlotinib PD (5 mo)

Legend: EGFRi, EGFR inhibitor; EGFR-KDD, EGFR kinase domain duplication; PR, partial response; SD, stable disease; PD, progressive disease; PFS, progression-free survival; NR, not reported;

In the largest multicentre study focused on clinical outcomes of EGFR-KDD to targeted therapy, Wang et al. reviewed 10,759 East Asian NSCLC patients who underwent NGS and identified 13 EGFR-KDD positive patients in total, of which 5 patients were treated with targeted therapy [68]. Two out of 5 patients did not respond to treatment with EGFRi including gefitinib, erlotinib and osimertinib and experienced disease progression with a short PFS of less than 3 months. The remaining 3 patients demonstrated either partial response to EGFRi for a minimum of 4 months with disease progression not yet reached at the time of publication, or stable disease for 11 months (summarised in Table 5). Collectively, these studies suggest that despite some heterogeneity in patient response, EGFR-KDD mutations are targetable by EGFRi. Additionally, both T790M mutation [68,69] and EGFR-KDD amplification [68,71] were reported in biopsies of EGFR-KDD tumour specimens post EGFRi treatment, suggesting shared resistance mechanisms with classical *EGFR* mutations to targeted therapy [72,73].

### 7.2 Complex mutations

In the context of *EGFR* mutations in NSCLC, the term “complex” or “compound” can refer to 3 possible scenarios: 1) classical L858R or Ex19Del *EGFR* mutations that also harbour additional *EGFR* mutations (classical + rare), 2) a combination of multiple, distinct, rare *EGFR* mutations that co-occur on the same allele (rare + rare), or 3) a combination of both L858R and Ex19Del *EGFR* mutations (classical + classical). Complex mutations have been reported account for ˜5 – 15% of *EGFR* mutations in NSCLC [65,[74], [75], [76]].

#### 7.2.1 Preclinical data

Performing *EGFR* exon sequencing on a cohort of NSCLC specimens, Kohsaka et al. report that over 90% of G719X mutations examined (*n* = 15) exist as complex mutations. Additionally, they report that over 75% of E709X mutations in the COSMIC database (*n* = 102) also exist as complex mutations [37]. Droplet Digital PCR (ddPCR) revealed that all compound mutations were present in *cis* alleles. Kohsaka et al. expressed a panel of rare mutations either alone or in combination in *trans* or in *cis* with L858R, G719A, G719C, or Ex19Del in NIH-3T3 and Ba/F3 cell line models. They found that NIH-3T3 cells expressing the complex mutations in *cis* formed more foci than either the single mutation or the complex mutations in *trans.* Taken together with the ddPCR data, this finding suggests that the transformation potential of complex mutations is higher than that of a rare *EGFR* mutation alone. The response of individual and complex *EGFR* mutations to gefitinib, erlotinib, afatinib and osimertinib were assessed using a high-throughput screen in which Ba/F3 cells expressing different mutations were uniquely barcoded and pooled together, with detection of barcodes by deep sequencing used to estimate relative cell numbers. For gefitinib, erlotinib and osimertinib treatment, the IC_50_ value for each complex *EGFR* mutation was intermediate between each corresponding single *EGFR* mutation. For example, the IC_50_ value for gefitinib treatment of L858R alone (4.4 nM) was lower than L858R + E709A (259 nM), but the complex mutation was more sensitive to drug treatment versus the single E709A rare mutation (785.8 nM). Interestingly, these differences were not observed for the irreversible second-generation EGFRi afatinib, which potently inhibited cell viability at similar levels across all single and complex *EGFR* mutations tested.

#### 7.2.2 Clinical data

*EGFR* complex mutations encompass a broad spectrum of different mutation combinations and as such a wide range of patient responses to EGFRi is expected. Nevertheless, with the exception of known resistance mutations such as T790M, complex *EGFR* mutations give rise to more favourable patient outcomes in response to EGFRi compared to single rare mutations alone [35,39,41,77]. In particular, the co-occurrence of a rare *EGFR* mutation with a classical L858R or Ex19Del mutation may be a strong indicator of sensitivity to EGFRi [40,78]. Baek et al. reported median PFS of 7.4 months for complex classical and rare mutation combinations following EGFRi treatment and 5.1 months for complex rare and rare mutation combinations. In both cases median PFS was significantly longer compared to patient groups of single rare exon 18 point mutations or exon 20 insertions (1.3 months and 2.6 months median PFS respectively) [78]. This supports preclinical data that demonstrate that complex mutations are more sensitive to EGFRi than single rare mutations [37]. Within rare and rare complex *EGFR* mutation combinations, it is likely that the sensitivity to EGFRi is influenced by the specific co-occurring partner mutation. For example, Chiu et al. observed differences in RR between G719X + L861Q (88.9%) versus G719X + S768I (50%) [39]. L858R + Ex19Del complex mutations achieved median 9.5 months PFS following EGFRi therapy [40], which is similar to the median PFS reported in patients with single L858R or Ex19Del mutations, indicating equivalent sensitivity of this class of complex mutations to the corresponding single mutation. Larger clinical studies informed by *in vitro* preclinical data will need to be conducted in order to predict the mutation combinations with the greatest sensitivity to EGFRi. Although patients with single classical *EGFR* mutations achieve the best clinical responses to EGFRi, these studies suggest that in most cases, patients with complex mutations show better clinical responses to EGFRi compared to patients with single rare *EGFR* mutations. In contrast, clinical responses are not as good for complex mutations that include a primary mutation that has been linked to resistance to first- or second-generation EGFRi, such as T790M. A poor RR of 8.3% and median PFS of 1.4 months was reported for patients that harboured complex *EGFR* mutations that contained either exon 20 insertions or T790M mutations [79].

## 8 Future perspectives

Structural data and preclinical models can shed light on the activation mechanisms of rare *EGFR* mutations and have been used to predict the sensitivity of different mutations to EGFRi. This information can translate into the identification of therapies that confer significant survival benefit over conventional chemotherapy when a sufficient patient population can be recruited and assessed in clinical trials, such as the successful approval of afatinib to treat patients harbouring rare G719X, S768I, or L861Q *EGFR* mutations [9]. The therapeutic landscape for classical *EGFR*-mutant NSCLC is rapidly evolving in response to the development of novel EGFRi, immunotherapy and rational drug combinations in order to determine the most effective and durable treatment strategy. However, there remain several outstanding issues that need to be addressed in order to ensure that research in rare *EGFR* mutations keeps apace with this fast-moving field.

### 8.1 Outstanding issues for rare *EGFR* mutations in NSCLC

Despite significant progress in our understanding of the mechanisms of receptor activation by rare *EGFR* mutations and the sensitivity of different mutations to EGFRi, little is known about the effect that different *EGFR* mutations have on the interactome and signalling networks downstream of EGFR. Neomorphic mutations within the same gene that display distinct signalling pathway dependencies have been observed elsewhere in cancer biology. For example, Cheung et al. noted mutation-specific drug sensitivities when studying different mutations in the *PI3KR1* gene, encoding the PI3K subunit p85α, which frequently occur across multiple cancer types [80]. Cheung et al. found that two mutations that occur in a similar region of the *PI3KR1* gene, the R348* truncation and L370^fs^ frameshift mutations, resulted in an increased sensitivity to MEK and JNK inhibitors compared to WT and mutations located elsewhere in the gene [80]. The authors show that R348* and L370^fs^ variants localize to the nucleus and provide a scaffold for components of the nuclear JNK pathway, conferring a unique vulnerability to disruption of MEK/JNK signalling pathways. These findings highlight the need to tailor targeted therapies not just to the gene which is mutated, but also toward the specific mutation within the gene. In the context of rare *EGFR* mutations in lung cancer, little is known about the differences in downstream signalling between distinct mutations. Mutation-specific patterns of downstream signalling activation have been observed when comparing EGFR WT with EGFR L858R and the EGFRvIII and EGFRvIV found in glioblastoma [[81], [82], [83]]. Future work will need to examine the interactome of different mutant receptors and profile the downstream signalling networks activated by different rare *EGFR* mutations. A deeper understanding of the biology of different *EGFR* mutations may reveal mutation-specific dependencies that could be exploited therapeutically through the use of combination strategies, polytherapies, or synthetic lethal approaches [84,85].

The rarity of the mutations described here has led to the field being frequently reliant on genetically engineered model systems. The use of model systems such as Ba/F3 cells means that any cancer cell-type specific effects are likely to be missed. To generate models which more closely match the original cancer context, techniques including TALEN or CRISPR have been recently used to genetically engineer lung cancer cell lines to replace EGFR WT or classical EGFR mutations with the desired rare EGFR mutations [53,55]. Such approaches allow the study of rare mutations in a cellular context that is more relevant to the clinical setting. Recently, a number of patient-derived cell lines and xenografts harbouring endogenous exon 20 insertion mutations have been generated [49,56,86]. These models have facilitated studies that have advanced our biological understanding of exon 20 insertion mutations, leading to the development of several compounds that target this class of mutations. Future work focusing on the development of cell lines harbouring other rare *EGFR* mutations from patients could lead to similar advancements in our understanding of rare *EGFR* mutations and how to treat them. Recent advances in generating patient-derived organoid models from NSCLC tumours can also be applied in this setting to evaluate targeted drugs and guide treatment decisions for rare *EGFR* mutations [87,88].

A major barrier to progress in the treatment of rare *EGFR* mutations is the lack of clinical trials that focus specifically on rare mutations. The clinical trials described in this review focus mainly on classical *EGFR* mutations, with the majority of clinical findings that specifically pertain to rare mutations coming from retrospective, multicentre analyses or post-hoc analyses of pooled clinical trials [9]. Moving forwards, it is important that patients with rare *EGFR* mutations are not excluded from larger trials to enable these types of post-hoc analyses that can determine the efficacy of EGFRi in the clinical setting. Increased uptake of NGS in routine diagnostics will help to identify additional patients with rare EGFR mutations that would be candidates for clinical trials. In particular, E709X mutations, EGFR-KDD and certain exon 20 insertions are not detected by commercially available PCR-based tests such as the current commercially available cobas® EGFR Mutation Test v2 platform (Roche). Given the heterogeneity of EGFRi response observed between different mutations and the influence of complex mutations on EGFRi sensitivity, caution must be taken to dissect the data to isolate mutation-specific effects. For example, although 100% RR was observed for afatinib in patients harbouring S768I mutations across LUX-Lung 2, LUX-Lung 3 and LUX-Lung 6 trials, 7 out of the 8 patients assessed harboured complex *EGFR* mutations [9,21,41,57]. Larger data sets are required to determine if afatinib remains effective in patients with S768I *EGFR* mutations alone. In an ideal setting, focused clinical trials that aim to recruit patients with rare *EGFR* mutations should be conducted to minimise patient heterogeneity. Several trials are already ongoing for patients with *EGFR* exon 20 insertions to assess EGFRi including osimertinib (NCT03414814), poziotinib (NCT03066206) [49], TAK-788 (NCT02716116) and tarloxotinib (NCT03805841). Similar trials for other rare *EGFR* mutations will help to refine therapeutic decisions based on mutation type.

### 8.2 Emerging treatment strategies for mutant EGFR NSCLC

There is growing evidence to support the use immunotherapies in the treatment of NSCLC, and several antibodies that target the PD1/PD-L1 immune checkpoint have shown significant PFS and OS benefits compared to chemotherapy in ˜20% of advanced NSCLC patients including pembrolizumab, nivolumab, durvalumab and atezolizumab [[89], [90], [91], [92], [93]]. However, a recent meta-analysis has highlighted that the same survival benefits with immunotherapies are not present in NSCLC patients with classical *EGFR* mutations [94]. Nevertheless, rare *EGFR* mutations have been associated with longer median PFS when treated with immune checkpoint inhibitors [95]. Retrospective analysis of *EGFR* mutant NSCLC patients treated with pembrolizumab or nivolumab revealed that patients harbouring G719X and exon 20 insertion mutations have longer median PFS compared to patients harbouring classical mutations (8.4 months vs 1.6 months; Yamada et al., 2019). Although encouraging, this study was conducted retrospectively using a small sample size (*n* = 27). Future clinical studies with larger sample sizes are required to validate the efficacy of immunotherapies for other rare *EGFR* mutations. Furthermore, it is necessary to establish whether immunotherapies are superior to available EGFRi such as afatinib. Yamada et al. report 8.4 months median PFS for patients harbouring either G719X or exon 20 insertion mutations treated with pembrolizumab or nivolumab compared to 13.8 months or 2.7 months median PFS for G719X or exon 20 insertions respectively when treated with afatinib [9,96,97]. These data suggest that immunotherapy may be a preferable therapeutic option for exon 20 insertion mutations, but not for G719X. Nonetheless, head-to-head trials comparing immunotherapies with EGFRi will be necessary to establish the usefulness of immunotherapies in the treatment of NSCLC with rare *EGFR* mutations.

A further obstacle to effective use of immunotherapies is the current lack of a predictive biomarker. Although PD-L1 expression has been identified as predictive of response in clinical trials in NSCLC, median OS following treatment with immunotherapy does not differ significantly with PD-L1 expression within the *EGFR* mutant group [89,90]. Similar findings have been reported in studies focusing specifically on rare *EGFR* mutations. A case report described 4 NSCLC patients with uncommon *EGFR* mutations and high PD-L1 expression (>50%) [98]. Three of the 4 patients described harboured G719X mutations, and were effectively treated with pembrolizumab. The remaining patient had a E746_T751delinsA + T790M compound mutation and despite having 60% PD-L1 expression did not respond to pembrolizumab treatment. Conversely, Gettinger et al. reported 2 out of 13 patients with *EGFR* mutations were alive at 5-year follow up of a phase I study of nivolumab in NSCLC, both of whom harboured rare mutations (G719A and exon 20 insertion mutation respectively) with low PD-L1 expression (<1%) [99]. The patient harbouring G719A showed a partial response with progressive disease 16 months after completion of the treatment protocol. Subsequent rechallenge with nivolumab resulted in a second partial response and 8 months PFS. The patient harbouring an exon 20 insertion mutation experienced 9 months PFS with nivolumab treatment. Overall these studies suggest that immunotherapies may be effective for certain rare *EGFR* mutations. However, they also demonstrate that the predictive value of PD-L1 overexpression as a biomarker for response to checkpoint inhibition in NSCLC with rare *EGFR* mutations remains unclear. To advance the use of immunotherapies in rare *EGFR* mutations, future work will need to focus on identifying a reliable predictive biomarker for response and establishing whether immunotherapies confer superior survival benefits compared to available EGFRi.

Another potential strategy is to combine EGFRi and immunotherapy to achieve a more durable response. A combination of EGFRi and immunotherapy *in vitro* has suggested that short-term erlotinib treatment is sufficient to enhance the susceptibility of NSCLC cell lines harbouring classical *EGFR* mutations to cell killing by cytotoxic natural killer cells and T cells, via an upregulation of caspase-mediated apoptosis [100]. This raises the exciting possibility that sequential or combinatorial EGFRi and immunotherapy could delay the onset of resistance to targeted therapies alone and enhance patient responses in the clinic. Several clinical trials are ongoing to assess the combination of EGFRi and immunotherapy either in the context of treatment-naïve or EGFRi-resistant NSCLC [101]. Although the data from these trials are not mature, minimal improvements have been observed for immunotherapy and EGFRi combination therapy versus EGFRi alone [102]. However, the combination treatment has thus far only been assessed in the classical *EGFR* mutation setting and is associated with a high degree of grade 3 and 4 toxicities. Thus, further investigational work is needed to clarify whether there is synergy between EGFRi and immunotherapy in patients, which patients are most likely to benefit, and what the optimal drug scheduling strategy will be.

Combination strategies involving conventional chemotherapy together with either EGFRi or immunotherapy are also being assessed in *EGFR* mutant NSCLC. A recent phase III trial found that addition of pemetrexed-carboplatin to gefitinib significantly prolonged median PFS in patients harbouring sensitizing mutations in exon 19, 21, or 18 compared to gefitinib alone (16 months vs 8 months; [103]). These exciting results raise the question of whether similar PFS benefits could be achieved in patients harbouring rare *EGFR* mutations that are sensitive to other EGFRi such as afatinib. Future clinical studies should assess the efficacy of pemetrexed-carboplatin in combination with afatinib for the treatment of patients harbouring G719X, S768I, or L861Q mutations.

Although combination strategies of immunotherapy and chemotherapy have shown efficacy for NSCLC patients without sensitizing *EGFR* mutations [104], the usefulness of these therapies in patients with *EGFR* mutant disease is unclear. While the IMpower150 trial found that combination of atezolizumab with carboplatin + paclitaxel + bevacizumab provided a significant median PFS benefit compared to the same treatment without atezolizumab in a patient group harbouring either *EGFR* or *ALK* mutations (9.7 months vs 6.1 months; [105]), the IMpower130 trial reported no benefit to patients harbouring *EGFR* mutations when treated with atezolizumab + chemotherapy [106]. It should be noted that the IMpower130 trial did not include bevacizumab, an anti-VEGF-A humanised monoclonal antibody, in the combination strategy, suggesting that bevacizumab may be important for activity in *EGFR* mutant patients. The median PFS benefit reported in the IMpower150 trial could be promising for the treatment of patients with rare *EGFR* mutations that do not respond to available EGFRi. However, both the IMpower150 trial and the IMpower130 trial excluded patients with *EGFR* mutations from their primary end-points. They also do not report the specific mutation in patients with *EGFR* mutations, meaning that no conclusions can be reached regarding the efficacy of immunotherapy – chemotherapy combinations in patients harbouring rare *EGFR* mutations. Future work focused on patients harbouring rare *EGFR* mutations will be required to assess the efficacy of immunotherapy – chemotherapy combinations in this setting. Another important caveat to the usefulness of immunotherapy – chemotherapy combinations is the increased toxicities associated with the combination strategy. The KEYNOTE-189 trial that assessed pembrolizumab in combination with chemotherapy reported discontinuation of treatment due to therapy related adverse events in 13.8% of patients receiving pembrolizumab - chemotherapy compared to 7.9% receiving placebo - chemotherapy [104]. Similarly, the KEYNOTE-407 trial reports discontinuation of treatment due to therapy related adverse events in 13.3% of patients receiving pembrolizumab - chemotherapy compared to 6.4% receiving placebo [107]. Establishing the safest approach to administering immunotherapy – chemotherapy combinations will be essential in advancing the use of these strategies in patients harbouring rare *EGFR* mutations.

## 9 Conclusion

Rare mutations in *EGFR* account for ˜15% of *EGFR* mutations in NSCLC, amounting to around 30,000 diagnoses per year owing to the high prevalence of lung cancer. Although many rare *EGFR* mutations are associated with poorer responses to first-generation EGFRi compared to classical *EGFR* mutations, more effective alternative EGFRi have been identified for several rare *EGFR* mutations such as exon 20 insertions. It is therefore essential that there is an increased uptake of improved detection methods such as NGS in clinical practice moving forward in order to identify rare *EGFR* mutations and assign the most effective EGFRi on a mutation-specific basis. The question of whether different rare *EGFR* mutations harbour differences in their interactomes or downstream signalling networks remains unanswered. In order to fully address this biological question it will be necessary to develop novel preclinical models of rare *EGFR* mutations. An improved understanding of the fundamental biology of different rare *EGFR* mutations may identify mutation-specific dependencies that could be exploited therapeutically. To address the paucity of clinical data pertaining to rare *EGFR* mutations, future studies must not exclude patients with rare *EGFR* mutations and should report PFS and OS data for each rare mutation separately to facilitate pooled post-hoc analyses. Clinical trials focusing specifically on rare *EGFR* mutations could significantly improve the treatment options for patients harbouring rare *EGFR* mutations.

Alternative strategies to EGFRi are also being assessed in *EGFR* mutant NSCLC. Immunotherapies have shown efficacy in patients harbouring rare *EGFR* mutations, potentially opening new treatment strategies for patients whose mutations do not respond well to available EGFRi. However, this exciting prospect is hampered by the small sample sizes in current studies and the lack of predictive biomarkers. Clinical trials with larger cohorts that focus on rare *EGFR* mutations and the identification of robust predictive biomarkers are essential for the advancement of these therapies in the clinic. Finally, combination of chemotherapy with gefitinib has led to longer median PFS in patients harbouring classical *EGFR* mutations. Future clinical trials assessing whether similar benefits can be achieved in patients with rare *EGFR* mutations that are sensitive to afatinib could significantly improve the treatment of patients with these mutations.
